# Systematic low-grade chronic inflammation and intrinsic mechanisms in polycystic ovary syndrome

**DOI:** 10.3389/fimmu.2024.1470283

**Published:** 2024-12-19

**Authors:** Hongxia Deng, Yan Chen, Jilong Xing, Nannan Zhang, Liangzhi Xu

**Affiliations:** ^1^ Reproductive Endocrinology and Regulation Laboratory, West China Second University Hospital, Sichuan University, Chengdu, China; ^2^ Key Laboratory of Birth Defects and Related Diseases of Women and Children, Ministry of Education, Sichuan University, Chengdu, China; ^3^ Department of Obstetrics and Gynecology, West China Second University Hospital, Sichuan University, Chengdu, China; ^4^ Division of Renal and Endocrinology, Qin Huang Hospital, Xi’an, China; ^5^ National Center for Birth Defect Monitoring, West China Second University Hospital, Sichuan University, Chengdu, China

**Keywords:** chronic inflammation, intrinsic mechanisms, intestinal microecological, steroid hormones, polycystic ovary syndrome

## Abstract

Polycystic ovary syndrome (PCOS) is a prevalent endocrine and metabolic disorder affecting 6-20% of women of childbearing age worldwide. Immune cell imbalance and dysregulation of inflammatory factors can lead to systematic low-grade chronic inflammation (SLCI), which plays a pivotal role in the pathogenesis of PCOS. A significant higher infiltration of immune cells such as macrophages and lymphocytes and pro-inflammatory factors IL-6 and TNF-α has been detected in PCOS organ systems, impacting not only the female reproductive system but also other organs such as the cardiovascular, intestine, liver, thyroid, brain and other organs. Obesity, insulin resistance (IR), steroid hormones imbalance and intestinal microecological imbalance, deficiencies in vitamin D and selenium, as well as hyperhomocysteinemia (HHcy) can induce systematic imbalance between pro-inflammatory and anti-inflammatory cells and molecules. The pro-inflammatory cells and cytokines also interact with obesity, steroid hormones imbalance and IR, leading to increased metabolic imbalance and reproductive-endocrine dysfunction in PCOS patients. This review aims to summarize the dysregulation of immune response in PCOS organ system and the intrinsic mechanisms affecting SLCI in PCOS to provide new insights for the systemic inflammatory treatment of PCOS in the future.

## 1 Introduction

Polycystic ovary syndrome (PCOS) is a prevalent reproductive endocrine and metabolic disorder affecting 6-20% of women of reproductive age globally (1). It is usually characterized by hyperandrogenism (HA), anovulation or oligo-ovulation, and polycystic ovary morphology (PCOM) (2). The detrimental effects of PCOS on fertility and long-term health in women have garnered considerable attention in the field of reproductive medicine. However, the etiology of PCOS remains unclear. Accumulating evidences suggested that PCOS might be a complex multigenic disorder, influenced by epigenetic and environmental factors, including lifestyle choices such as diet, exercise, rest, tobacco and alcohol consumption, psychological stress, and exposure to various pollutants like environmental endocrine disruptors (3, 4). In addition to insulin resistance(IR) and compensatory hyperinsulinemia, PCOS patients experience an elevated risk for various metabolic disorders and malignancies in the long term (5, 6). Disruptions in common regulatory mechanisms between substance-energy metabolism and reproduction may contribute to concurrent metabolic and reproductive disorders, which aligns with the evolutionary understanding of the disorder (7).

Systematic low-grade chronic inflammation (SLCI) plays a pivotal role in the pathogenesis of various chronic disorders, including PCOS. An imbalance between immune cells and inflammatory cytokines is evident in the serum, ovaries and organs of PCOS patients (8). The interplay between inflammatory state and obesity, HA and IR, leads to increased metabolic imbalance and reproductive-endocrine dysfunction in PCOS patients (9). Furthermore, SLCI contributes to PCOS-related complications of multi-organ dysfunction, including cardiovascular diseases (CVDs), non-alcoholic fatty liver disease (NAFLD), and depression (10–12). Therefore, a comprehensive understanding of SLCI in PCOS is crucial for effective prevention and management strategies.

**Figure 1:**
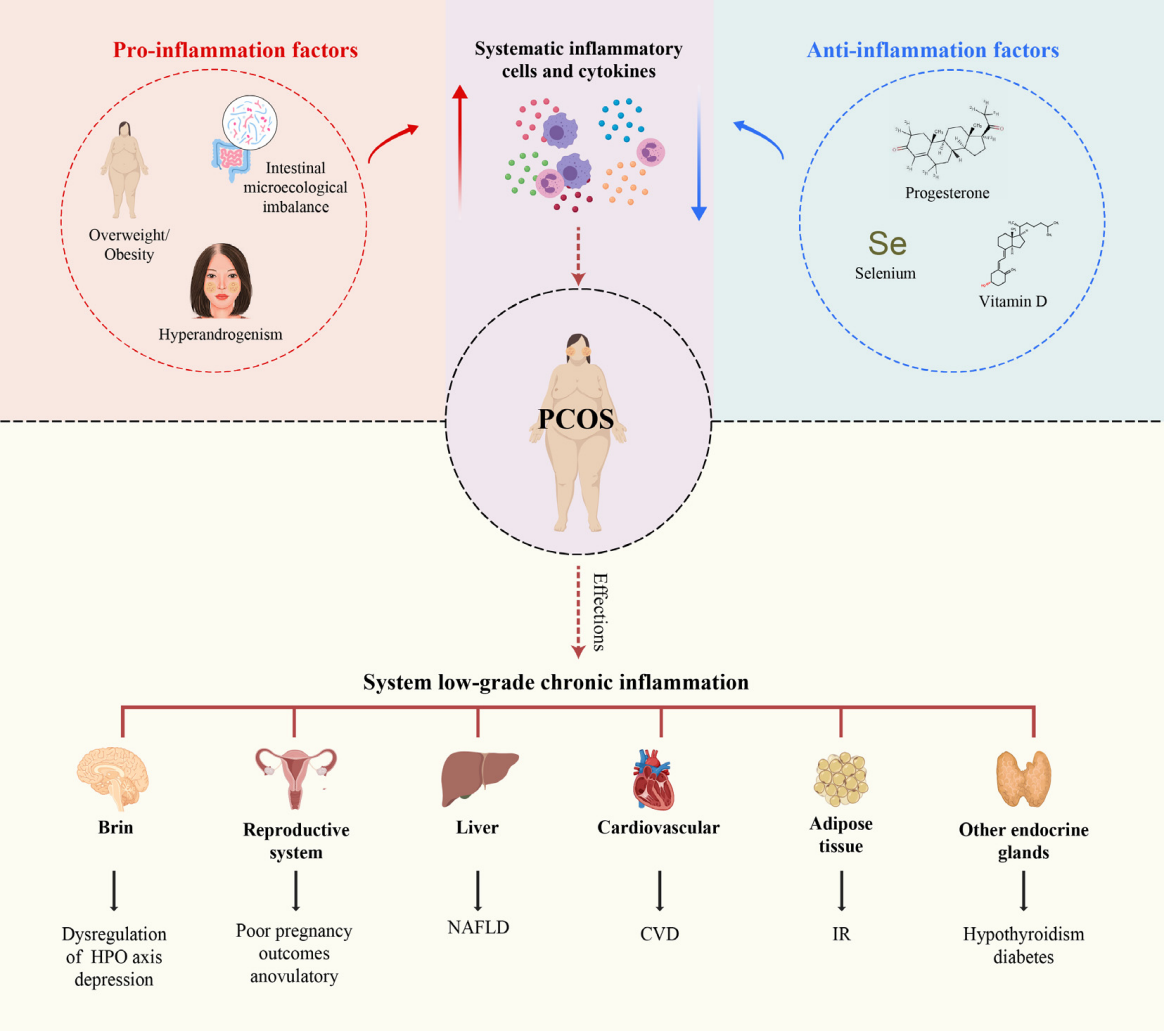
Immune dysfunction in PCOS and the intrinsic mechanisms influencing SLCI in PCOS. Immune dysfunction in PCOS affects not only the female reproductive system but also other organ systems, including the cardiovascular, intestinal, hepatic, thyroid, pancreatic, adrenal gland,brain and other organs. Obesity, IR, hyperandrogenism, and intestinal microecological imbalance, and hyperhomocysteinemia induce the secretion of systematic pro-inflammatory cells and cytokines. Conversely, deficiencies in progesterone, vitamin D, and selenium have the exert opposite effects.

This review elucidates the critical role of SLCI in the development of PCOS by analyzing the systemic mechanisms of chronic inflammation in PCOS from a novel perspective on the disruption of the balance between pro-inflammatory and anti-inflammatory factors. This review aims to deepen our understandings and provide new insights into the pathogenesis and treatment of PCOS (**Figure 1**).

## 2 SLCI in PCOS

**Figure 2:**
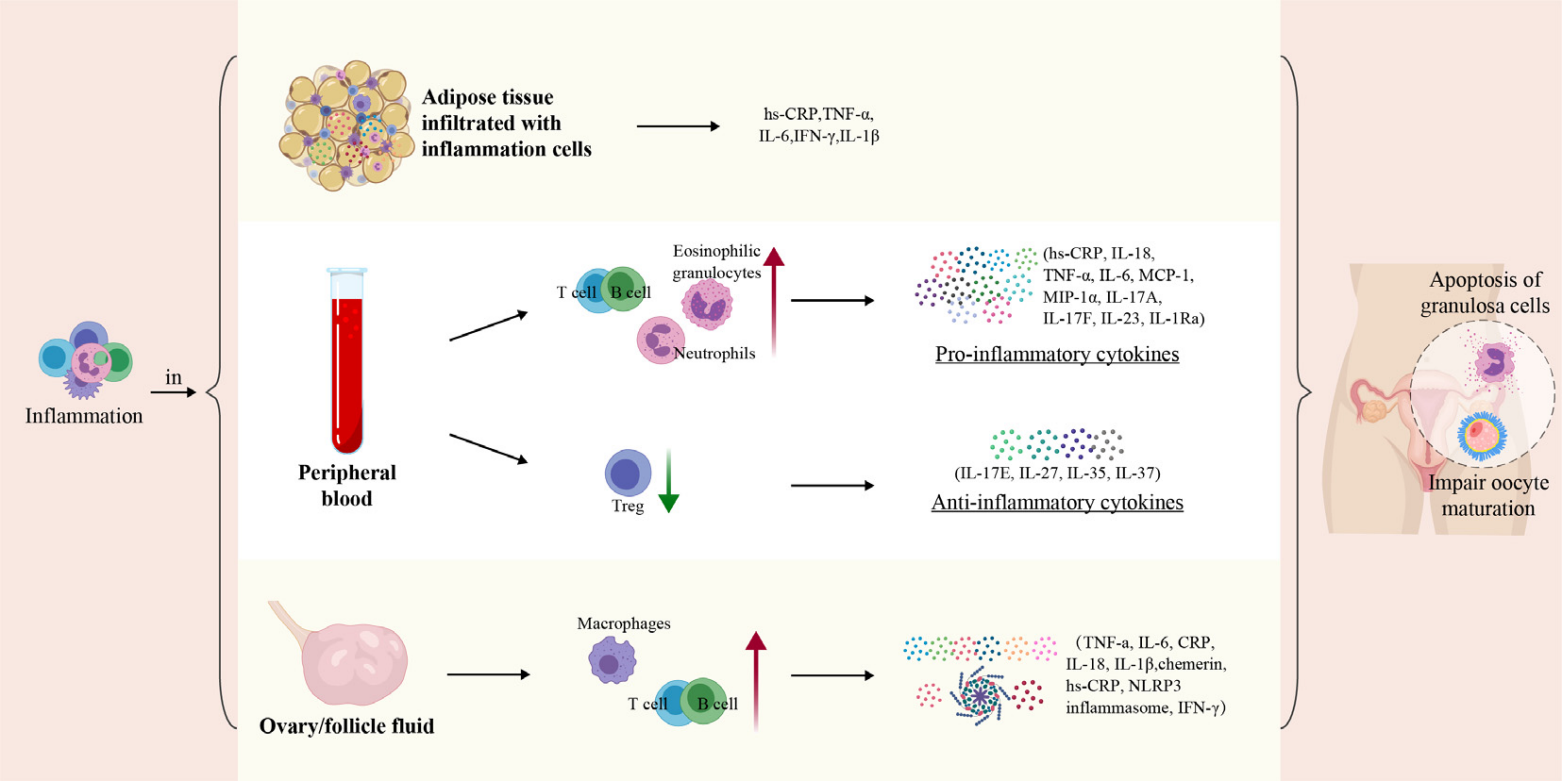
In women with PCOS, there is a discernible imbalance in the inflammatory cells and cytokines in the peripheral blood, FF, ovaries, and adipose tissue. Inflammatory mediators interact with factors such as obesity, IR, hyperandrogenism, and intestinal microecological imbalances. This interplay may exaggerate the pathophysiological features of PCOS. In adipose tissues, peripheral blood, ovary/follicle fluid of PCOS, immune cells such as T cells, B cells, and neutrophils are activated, leading to the secretion of inflammation cytokines such as TNF-α, MCP-1, IL-17, IL-23 and IL-18. Subsequently, this inflammatory milieu may induce apoptosis in the ovarian GCs and impair oocyte maturation, contributing to the reproductive dysfunctions observed in PCOS.

SLCI in PCOS patients plays a crucial role in disease progression (9). Compared with age-matched healthy women (controls), PCOS patients exhibited significantly increased amounts of immune cells, such as lymphocytes, neutrophils, monocytes, macrophages, and eosinophilic granulocytes in the peripheral blood (13–17). Additionally, elevated levels of inflammatory factors, such as high sensitive C-reactive protein (hs-CRP), interleukin-18 (IL-18), tumor necrosis factor α (TNF-α), interleukin-6 (IL-6), monocyte chemoattractant protein-1 (MCP-1), and macrophage inflammatory protein-1α (MIP-1α), are detected in the peripheral blood of PCOS patients (9, 18), indicating the presence of SLCI (**Table 1**). A significantly higher infiltration of immune cells, such as macrophages and lymphocytes, were detected in the ovaries of PCOS patients. Macrophages and immature dendritic cells (iDCs) were elevated in the endometrial tissue, and macrophages were also identified in the hearts of PCOS mice. Macrophages, neutrophils, mast cells, B cells, T cells, NKT cells infiltrate the adipose tissue. Additionally, inflammatory factors such as TNF-α, IL-6, CRP, IL-12, IFN-γ, and NLRP3 inflammasome were elevated in various tissues, including those in the ovaries and follicular fluid (FF), adipose tissue, endometrium, intestine and liver (8, 15, 17, 33). Excessive secretion of inflammatory factors TNF-α and IL-6 by immune cells activates the inflammatory signaling pathways, leading to cell damage and fibrosis within interstitial cells, ultimately causing dysfunction in these organs systems. Chronic immune response dysregulation contributes to PCOS-related immune dysregulation in the female reproductive system, cardiovascular system, cardiovascular, digestive, and endocrine systems (8). Correlation analysis indicated that increased serum inflammatory cytokine levels were strongly related to the severity of obesity, IR, ovulation disorder, and HA in PCOS patients (9, 34, 35). Taken together, the systematic imbalance between pro-inflammatory and anti-inflammatory factors in PCOS patients gives rise to the PCOS phenotypes. The pivotal role of the chronic inflammation in these organs systems and the intrinsic mechanisms of PCOS have been elaborated in the subsequent sections (**Figure 2**).

**Table 1:** Inflammatory markers which are the most predictive in the serum of PCOS patients.

Inflammatory markers	Years	Study	Tendency	Role	References
IL-6	2020	Review	Increase	The increase of IL-6 in the ovary can reduce the conversion of androstenedione to estradiol by inhibiting the aromatase activity of granulosa cells (GCs), leading to excessive androgen production	(19)
CRP	2019	Editorial	Increase	The levels of CRP in PCOS patients are significantly higher, regardless of whether they have a low body mass index (BMI) or are obese. This suggests that CRP may be a marker for identifying the risk of future cardiovascular diseases (CVDs) in young women with PCOS.	(20)
TNF-α	1997	Review	Increase	TNF‐α inhibits steroidogenesis of the thecal cells and GCs leading to regression of corpus luteal	(21)
IL-18	2004	Clinical study	Increase	The serum levels of IL-18 in the PCOS group were significantly higher. Furthermore, PCOS patients with insulin resistance (IR) and obesity had higher serum IL-18 levels, suggesting that IR and obesity may accelerate the increase in serum IL-18 levels. The study also found that IL-18 was positively correlated with BMI, IR, and testosterone (T).	(22)
WBC	2015	Clinical study	Increase	PCOS patients had significantly higher WBC counts, which were positively correlated with BMI, total T, insulin, triglyceride (TG), homeostasis model assessment (HOMA) scores, free androgen index(FAI), and sex hormone-binding globulin(SHBG), and negatively correlated with high-density lipoprotein(HDL). Multiple regression analysis showed that BMI, SHBG, and TG were the main predictive factors for WBC in PCOS.	(23)
IL-15	2022	Research and clinical study	Increase	IL-15 is involved in the pathogenesis of PCOS potentially by affecting survival, the inflammation state and steroidogenesis of GCs.	(24)
IL-17a	2020	Clinical study	Increase	The levels of IL-17a are significantly higher in PCOS patients. Its original negative correlation with anti-Müllerian hormone (AMH) levels is altered, thereby weakening glycolipid metabolism and promoting IR	(25)
IL-1Ra	2017	Clinical study	Increase	PCOS patients have significantly higher levels of IL-1Ra, which may reduce IR and glucose metabolism, leading to obesity and metabolic syndrome.	(26)
NLR	2022	Systematic review and meta-analysis	Increase	PCOS have a significantly increased NIR, which was significantly positively associated with fasting blood glucose and total cholesterol levels in PCOS	(27)
MPV	2022	Systematic review and meta-analysis	Increase	PCOS have a significantly increased MPV than women without PCOS, which is probably associated with IR	(28)
IL-22	2023	Review	Decrease	IL-22 has been shown to be therapeutically effective in immunological dysfunction and metabolic diseases, which suggests a role in the treatment of PCOS	(29)
AGEs	2005	Clinical study	Increase	A positive correlation was also observed between AGE proteins and the free androgen index (FAI), waist-to-hip ratio (WHR), insulin, HOMA	(30)
MCP-1	2021	Meta-analysis	Increase	It revealed that the circulating levels of MCP-1 are upregulated in women with PCOS and are associated with an increased risk of PCOS	(31)
CRP/albumin	2024	Clinical study	Increase	The CRP/albumin ratio was found to be significantly higher in women with PCOS as compared to healthy controls along with serum total testosterone and HOMA-IR	(32)

IL-interleukin, CRP-C-reactive, TNF-α-tumor necrosis factor α, WBC-White Blood Cell Count, IL-1Ra-interleukin-1 receptor antagonist, NLR-Neutrophil-to-lymphocyte ratio, MPV-mean platelet volume, AGEs-Advanced Glycation End-Products, INF-INF-gamma, MCP-1-monocyte chemoattractant protein-1, MIP-1a-macrophage inflammatory protein-1α, CRP/albumin-CRP albumin ratio.

### 2.1 Reproductive system inflammation in PCOS

#### 2.1.1 Ovary

SLCI contributes to defects in oocyte quality, leading to ovulatory infertility, and accelerating the decline in ovarian reserve (35). Increased inflammatory response has been observed in the ovarian tissues of both PCOS patients and PCOS-like rodent models (15). Histological analysis comparing ovarian samples from of 53 PCOS patients and 48 healthy controls revealed a higher infiltration of macrophages and lymphocytes in PCOS patients. These cells secrete excessive amounts of pro-inflammatory cytokines such as TNF-α and IL-6 (9). Furthermore, inflammatory mediators such as IL-18, IL-18-binding protein (IL-18BP), pentraxin3 (PTX3), IL-1β, chemerin, hs-CRP, IFN-γ, and TNF-α were elevated in the FF from PCOS patients undergoing *in vitro* fertilization and embryo transfer (IVF-ET) treatment, reflecting the local function and pathological state within the PCOS ovaries (9, 36, 37). Macrophages and lymphocytes infiltration have also been detected in PCOS-like mice (38). PCOS-like rodent models exhibited increased levels of TNF-α, IFN-γ, NLRP3 and caspase-1within the ovaries (38, 39). These immune perturbations contribute to reduced fertility and metabolic comorbidities in PCOS-like models and PCOS patients. However, PCOS-like models may not fully replicate the complexity of PCOS in humans, particularly in terms of inflammatory markers and their expression levels. In PCOS patients, inflammation is influenced by factors such as obesity and IR which may not be identically represented in animal models of PCOS.

Pro-inflammatory TNF-α, primarily secreted by macrophages, was significantly increased in the ovaries and FF of PCOS patients (40, 41). TNF-α and its receptor TNFRI (TNF-a receptor 1) are expressed in mammalian oocytes and surrounding granulosa cells (GCs), indicating a direct impact of TNF-α on cells involved in reproductive function (42–44). *In vitro* experiments demonstrated that TNF-α can directly induce apoptosis of primary rat GCs after 24h of incubation in serum-free medium 24 (45). Furthermore, *in vivo* treatment with TNF‐α inhibits steroidogenesis of the thecal cells and GCs, leading to regression of the corpus luteal (21).

Another pro-inflammatory cytokine IL-6, which is produced by mononuclear (MNC) cells and adipose tissue cells. The levels of IL‐6 in the peripheral blood, ovaries, and FF in PCOS patients were higher than those in healthy control women (15, 46). IL-6 may serve as an early low-grade chronic inflammatory marker in PCOS patients with IRS-2 polymorphism (47). Higher IL-6 levels in the peripheral blood of both lean and obese women with PCOS were significantly associated with homeostasis model assessment of insulin resistance (HOMA2-IR) ratio and total testosterone ratios (48). Increased IL-6 levels in the ovary can reduce the conversion of androstenedione to estradiol by inhibiting the aromatase activity of GCs, leading to excessive androgen production (19, 49).

Inflammatory cytokines IL-1β and IL-18 present in the ovarian tissue and FF in PCOS patients alter the follicular microenvironment by binding to receptors like IL-1R and TLR4 on the GCs, activating NF-κB, which subsequently translocates into the nucleus (50). Activated NF-κB promotes gene expression of key components of the NLRP3 inflammasome, such as NLRP3, ASC and caspase-1, resulting in the death of GCs and inhibition of oocyte maturation, eventually disrupting ovarian function (50).

These results suggested that the increased pro-inflammatory profile in the ovary and FF mediates apoptosis of GCs, leading to excessive follicle atresia. This stimulates the production of excessive testosterone by thecal cells, disrupting the hypothalamic-pituitary-ovarian (HPO) axis, ultimately affecting dominant follicle generation, follicular dysfunction, and ovarian interstitial cell fibrosis (51, 52).

#### 2.1.2 Uterus

Successful pregnancy, encompassing embryo adhesion, implantation, growth, invasion of trophocytes, and formation and functional maintenance of the placenta, requires precise regulation of immune cells and inflammatory factors at the maternal-fetal interface to establish immune tolerance (53). This immune and inflammatory regulation in the endometrial tissue, also known as endometrial receptivity (ER), refers to the compatible state between the embryo and maternal uterus (54, 55). Reduced ER contributes significantly to early pregnancy loss, often resulting from dysregulation of immune and inflammatory responses in the endometrial tissue observed in women with autoimmune diseases (AID) or antiphospholipid syndrome (APS). Notably, the prevalence of recurrent miscarriages during early pregnancy due to an abnormal ER is higher in PCOS patients (56).

Immunohistochemical analysis of endometrial tissues from PCOS patients revealed an increase in the presence of endometrial inflammatory cells, including macrophages, iDCs, mature dendritic cells (mDCs), and CD8^+^ T cells (33), indicating increased chronic inflammation in the endometrial tissues. Additionally, PCOS endometrial stromal fibroblasts produce higher levels of IL-6, IL-8, MCP-1, and granulocyte-macrophage colony-stimulating factor (GM-CSF), which facilitate the maturation of endometrial DCs and macrophages, subsequently leading to progesterone resistance in the endometrium and impairing endometrial decidualization (57).

The NLRP3 inflammasome, which plays a crucial role in the processing of pro-IL-1β and pro-IL-18 into their mature forms through caspase-1, has been significantly increased in the endometrium of women with recurrent miscarriage (58), suggesting a potential dysregulation of ER mediated through inflammasome function. Therefore, NLRP3 may serve as a novel biomarker of ER dysfunction. As expected, increased expression of NLRP3 in the endometrial tissue has been implicated in the pathogenesis of higher miscarriage rates in PCOS patients (18, 59). In contrast, stanniocalcin-1 (STC-1), a glycoprotein, known for its ability to mitigate inflammatory stress is reduced in the endometrium of PCOS patients (60). This diminished expression of STC-1 may contribute to a weakened protective response to inflammatory stress (61). These findings suggested that the decrease in ER observed in PCOS is due to an imbalance between pro-inflammatory and anti-inflammatory cells and factors.

In addition to decreased ER, which contributes to adverse pregnancy outcomes, the chronic inflammation may also be associated with an increased long-term risk of endometrial carcinogenesis in PCOS patients. Simultaneously, the expression of both inflammation-related genes (CCL-2, IL-6, TNF-α, induced protein 6 [TNFAIP6] and pro-oncogenic genes (cell adhesion molecule with homology to L1CAM [CHL1]) is upregulated in the endometrium of PCOS patients than in controls (62). These findings highlight the importance of understanding and addressing the immunological and inflammatory aspects of PCOS to improve pregnancy outcomes and reduce long-term health risks for patients.

### 2.2 Other endocrine glands inflammation in PCOS

#### 2.2.1 Thyroid gland

Autoimmune thyroid disease (AITD) is a common autoimmune disorder. PCOS and AITD share several clinical symptoms, including menstrual irregularities, infertility, obesity, IR, and dyslipidemia (63, 64).Therefore, screening for thyroid function and thyroid-specific autoantibodies is often recommended in the clinical diagnosis of PCOS. Furthermore, PCOS and AITD exhibit a strong clinical association. The prevalence of AITD in PCOS patients was significantly higher than that in the non-PCOS patients (65–68). Additionally, levels of thyroid-related autoantibodies, such as anti-TSH, anti-TPO, and anti-Tg, as well as T- and B-cell infiltration into the thyroid gland were higher in PCOS patients than controls (69–71). The elevated androgen levels in PCOS patients may contribute to AITD. Excessive androgens enhance the activity of T suppressor cells or promote Th1 responses, and Th1-mediated autoimmunity, resulting in thyroid cytolysis and hypothyroidism (68). Furthermore, PCOS patients exhibit compensatory increases in estrogen levels and inadequate progesterone levels. Estrogen may upregulate IL-6 expression in T cells, whereas inadequate of progesterone suppression potentially results in immune system overactivation (72). These findings indicates that the disruption of steroid hormones in PCOS patients contributes to inflammation dysregulation in the thyroid gland, leading to thyroid dysfunction. In summary, the clinical overlap between PCOS and AITD, along with the evidence of immune system dysregulation and thyroid dysfunction in PCOS patients, underscores the importance of considering thyroid health in the management of PCOS.

#### 2.2.2 Pancreas

Pancreatic β-cell dysfunction is prevalent in PCOS patients, which significantly contributes to their abnormal glucose tolerance and the long-term risk of developing type II diabetes mellitus (T2DM) (73, 74). Malin et al. reported a direct relationship between β-cell dysfunction in PCOS patients and the MNC-derived NF-κB activation as well as an inverse correlation with IκB expression, indicating impaired inflammation regulation in the pancreas of PCOS patients (75). Conversely, nanocurcumin, a potential anti-inflammatory agent, significantly reduced oxidative markers and TNF-α levels in the pancreas, alleviated IR, restored islets integrity in PCOS models (75–77). Additionally, medications such as saxagliptin and metformin have demonstrated effectiveness in regulating β-cell function by reducing inflammation in newly diagnosed T2DM patients with PCOS (74).These findings suggest that the crucial role of pancreatic inflammation and β-cell dysfunction in PCOS-related metabolic complications and highlight the need for targeted interventions that can improve β-cell function and reduce inflammation.

#### 2.2.3 Adrenal gland

Adrenal immune damage has recently been identified in patients with PCOS, coinciding with the overproduction of adrenal androgens(AA) (78, 79). Dehydroepiandrosterone (DHEA) and its sulfated form, dehydroepiandrosterone sulfate (DHEA-S), are the predominant AA in PCOS patients. DHEA synthesized in the adrenal gland is converted into DHEA-S and released into the bloodstream. Circulating DHEA-S levels has been used as a maker of adrenocortical dysfunction in PCOS (79). DHEA plays a role in maintaining ovarian immune homeostasis by modulating the balance between Th1 and Th2 immune responses within the ovary through NF-κB regulation, which decreases IL-2 and IL-10 (80). DHEA-S exerts immunomodulatory effects, reducing the T cell population with a concurrent increase in NK and T cells. These findings suggest a potential association between AA and immunological response in PCOS.

However, a retrospective cohort study identified a previously unrecognized infertile PCOS-like phenotype characterized by elevated levels of anti-Müllerian hormone (AMH) and low total testosterone, DHEA-S, and cortisol. Notably, this phenotype is also associated with increased levels of thyroid autoimmunity markers, such as thyroid autoimmunity (TPO antibodies) and the inflammatory markers CRP and IL-6. Therefore, this hypo-androgenic PCOS phenotype (HH-PCOS) may be related to the autoimmune damage in the adrenal zona reticularis (81, 82). However, further research is needed to confirm chronic inflammation in the adrenal glands and its potential effects on endocrine function in PCOS patients. These findings revealed a complex interplay between AA overproduction and immune dysfunction with adrenal gland in PCOS, Additionally, they indicate that infertile PCOS-like phenotype may be an immunoinflammatory disorder associated. This underscores the importance of reducing the AA levels and inflammation to ameliorate HH-PCOS.

#### 2.2.4 Hypothalamus

Inflammatory markers, including IL-1β, IL-6, and TNF-α, were significantly upregulated within the hypothalamus of PCOS-like rats, indicating the presence of chronic low-grade neuroinflammation (83, 84). Hypothalamic inflammation contributes to the occurrence and progression of numerous metabolic disorders in PCOS, including obesity, diabetes, hypertension, and dyslipidemia, by affecting food intake homeostasis, energy balance, insulin and leptin signaling, glucose metabolism and fatty acid oxidation in the liver (85). High-fat diet (HFD)-induced overactivation and/or excessive M1-type macrophages-microglia, which is characterized by a pro-inflammatory response in the central nervous system. This M1-polarized microglia induce an inflammatory response and release a large number of inflammatory factors, such as nitric oxide, IL-6, TNF-α, and reactive oxygen species (ROS), which are key drivers of hypothalamic inflammation (86–89). Furthermore, hypothalamic inflammation alters the pulsatile secretion pattern of gonadotropin-releasing hormone (GnRH), resulting in an increase in luteinizing hormone (LH)/follicle stimulating hormone (FSH) ratios and leading to irregular menstrual cycles and ovulatory disorders (90). These findings highlight the strong association between hypothalamic inflammation and the etiology and phenotype of PCOS, suggesting that targeting hypothalamic inflammation could be a potential therapeutic strategy (91).

### 2.3 Inflammation of non-endocrine organs in PCOS

#### 2.3.1 Cardiovascular system

The risk of CVDs is higher in PCOS patients (92–94). Vascular alterations, such as endothelial dysfunction, increased arterial stiffness, enhanced intima-media thickness, and arterial wall calcification, are prevalent even in young women with PCOS (94–96). Furthermore, vascular endothelial injury and endothelial cell dysfunction in PCOS patients are independent of age, body weight, and metabolic abnormalities, suggesting that PCOS may be an independent risk factor for CVDs and could lead to an earlier onset of CVDs despite the presence of metabolic disorders (97, 98).The current consensus is that chronic inflammation of the vascular endothelium and the resulting endothelial dysfunction are fundamental to the pathogenesis of CVDs (99, 100). Additionally, pro-inflammatory factors such as CRP, IL-6 and IL-18, which are closely associated with the incidence of CVDs, are also elevated in the plasma of women with PCOS patients (18).

The critical role of macrophages in the pathogenesis of CVDs in PCOS has been recently reported, with a substantial augmentation in the number of M1-macrophages in the hearts of PCOS model mice, predominantly derived from circulating monocytes (10). Furthermore, PCOS mice with atherosclerosis and myocardial infarction exhibited a pronounced infiltration of macrophages into the myocardium (10). The adverse cardiovascular effects of PCOS could be attributed to the over-activation of the norepinephrine-NF-κB pro-inflammatory signaling pathway, contributing to increased expression of a hematopoietic progenitor retention factor, vascular cell adhesion molecule 1(Vcam1), in splenic macrophages, subsequently resulting in increased circulating total monocytes and inflammatory monocytes. These findings indicate that the accumulation of macrophages in the heart contributes to endothelial dysfunction, emphasizing the need to address chronic inflammatory state in PCOS patients for effective management of their cardiovascular health (10).

#### 2.3.2 Liver

The prevalence of NAFLD is higher in PCOS patients than in healthy women (34%–70% vs. 14%–34%) (101, 102). IR, obesity, HA, chronic inflammation, genetic factors and dyslipidemia are risk factors for NAFLD development in PCOS patients (103–105). Interestingly, HA in PCOS is an independent risk factor for NAFLD, as demonstrated in a recent systematic review and meta-analysis (101, 106). Bioinformatics data identified 52 differentially expressed genes (DEGs) shared between PCOS and NAFLD. Gene ontology (GO) and Kyoto Encyclopedia of Genes and Genomes (KEGG) pathway enrichment analyses suggested that these DEGs were mostly enriched in immunity- and inflammation-related pathways (107). NAFLD-like hepatic pathological changes such as steatosis, inflammatory cell infiltration, necrotic hepatocytes, and liver fibrosis, accompanied by increased expression of inflammatory cytokines (TNF-α, IL-1β), stress-related protein urocortin-1, antioxidant gene glutathione peroxidase-1 (Gpx1), and the NLRP3 inflammasome within the hepatic tissues, have been observed in several rodent models of PCOS (108–111). Over-production of these pro-inflammatory mediators in the liver of individuals with PCOS leads to the infiltration of various immune cells, including macrophages, T lymphocytes, dendritic cells, and neutrophils (11). These cells further release additional inflammatory cytokines that interact with adipokines, such as leptin, adiponectin, vaspin, visfatin, and chimerin. This exacerbates the imbalance between the pro-inflammatory and anti-inflammatory states within the liver, ultimately contributing to the development of the NAFLD in PCOS (12).

#### 2.3.3 Lung

The association between PCOS and inflammatory diseases of the lung has not been extensively investigated. Recent studies on the association between PCOS and coronavirus-induced disease 19 (COVID-19) have provided valuable insights. COVID-19 is characterized by an excess of pulmonary inflammatory cells such as macrophages, neutrophils, and dendritic cells and elevated levels of inflammatory factors, including IL-2, IL-7, IL-10, G-CSF, IP-10, MCP-1, MIP1A, and TNF-α. Overproduction of these factors can ultimately lead to damage and death of alveolar epithelial cells, potentially leading to respiratory failure and death (112). PCOS patients are more susceptible to infection by severe acute respiratory syndrome coronavirus 2(SARS-CoV-2) and exhibit more severe clinical symptoms than those of control women without PCOS (113). PCOS patients exhibit higher infection rates (28%–50%) across all age groups, leading to increased hospitalization rates and morbidity and mortality rates than those of age-matched women without PCOS. HA may be a factor in the increased susceptibility to SARS-CoV-2 in PCOS in individuals with PCOS (114). In dihydrotestosterone (DHT)-induced PCOS-like female mice, DHT up-regulates ACE2 mRNA in the lung, cecum, heart and kidney, which synergizes with host transmembrane protease serine 2 (TMPRSS2) to facilitate SARS-CoV-2 viral entry into the host cells (115). These findings suggest that PCOS patients, particularly those with HA, are more susceptible to COVID-19, which leads to increased pulmonary inflammation and damage in the lung tissues, ultimately contributing to an increased mortality rate.

#### 2.3.4 Brain

Approximately 5%-10% of reproductive-age women without PCOS worldwide experience depression and anxiety, and 40% of women with PCOS suffer from depression (1, 116). Psychiatric disorders, including generalized depressive disorder, are inflammatory conditions characterized by elevated levels of inflammatory markers (117–119). Inflammatory markers such as CRP, IL-1, IL-6 and TNF-α elevated both in patients with depression and PCOS (120, 121). Inflammatory factors may penetrate the blood-brain barrier (BBB) via cytokine-specific transport mechanisms, potentially involving the active transport of saturable transporter molecules (122, 123). Subsequently, increased inflammatory factors can disrupt the metabolism of brain monoamines such as neuronal 5-hydroxytryptamine (5HT) and dopamine (DA), which are hypothesized to contribute to the pathogenesis of depressive disorders (124–126). Nevertheless, experimental animal studies to elucidate the mechanisms by which inflammatory mediators induce depressive disorders in PCOS are lacking. The complex relationship between PCOS and psychiatric disorders, particularly depression and anxiety, underscores the need for further research to better understand and address these comorbidities.

## 3 Intrinsic mechanisms affecting SLCI in PCOS

### 3.1 Obesity and IR in PCOS

The clinical comorbidities associated with metabolic disorders in PCOS include obesity and IR. Approximately 52%-64% of women with PCOS are either overweight or obese, which is an independent risk factor for IR in PCOS. Additionally, approximately 30% of PCOS patients with a normal body mass index (BMI) also show abdominal obesity, marked by an excessive accumulation of visceral fat (127, 128). Obesity also contributes to ovarian inflammation, steroidogenesis, and ovulation (129). Visceral obesity in PCOS patients primarily contributes to systemic inflammation throughout the body. The infiltration of immune cells such as macrophages, neutrophils, mast cells, B cells, T cells, NKT cells within the adipose tissue, coupled with the overproduction of inflammatory mediators and adipokines (e.g., leptin and lipocalin), adipocyte-derived MCP-1 and hypertrophy of visceral adipocytes, leads to hypoxia, autophagy, and apoptosis (17, 130). Additionally, hypoxia adipocytes can activate the c-Jun N-terminal kinase (JNK) and NF-κB pathways, resulting in the synthesis and secretion of pro-inflammatory factors such as IL-6, TNF-α, IL-1β, IL-12, and IFN-γ, which induce a SLCI state in adipose tissue (131, 132). Specifically, adipose tissue-resident macrophages exacerbate IR by elevating the levels of TNF-α, which in turn increase the phosphorylation of insulin receptors substrate-1 (IRS-I) (phospho-IRS-I). This event, through the phosphatidylinositol 3-kinase (PI3K) pathway, which inhibits the activation of protein kinase B (PKB), a pivotal enzyme regulating the insulin-sensitive glucose transporter type 4 (GLUT-4), ultimately resulting in IR (133, 134). These additional release mediators released into the circulation can induce inflammatory responses in extra-adipose tissues, including the ovaries and peri-ovarian adipose tissues (129, 135).

IR is present in 50%-70% of PCOS patients, and high insulin levels in the FF potentially directly stimulate the LH receptors on thecal cells, increasing their sensitivity to LH or reducing the hepatic production of sex hormone binding globulin (SHBG). Elevated free testosterone levels lead to impaired follicle development in PCOS patients (136). In PCOS patients with IR, the underlying mechanisms involves a dysfunction of the PI3K pathway, whereas the MAPK pathway remains functional (137–139). Impaired mitochondrial function due to the downregulation of nuclear-encoded genes involved in oxidative phosphorylation increases ROS production, which in turn phosphorylates the serine residues of insulin receptors and IRSs, thereby resulting in SLCI and a reduction in IR. Consequently, these processes synergistically contribute to the exacerbation of SLCI in PCOS.

Obesity and IR can exacerbate the SLCI in PCOS. A causal relationship between obesity, IR, and increased pro-inflammatory activity within adipose tissue. In summary, the molecular mechanisms associated with obesity and IR play a pivotal role in the development of the SLCI in PCOS (140).

### 3.2 Imbalance of the sex hormones in PCOS

#### 3.2.1 Androgen

The primary etiology and symptoms of PCOS are predominantly associated with the dysregulation of steroid hormones, particularly elevated levels of androgen and decreased progesterone levels linked to luteal phase deficiency. Both androgens and progesterone play crucial roles in the inflammation response. As a pro-inflammatory steroid hormone, HA induces SLCI in the ovaries by stimulating monocyte infiltration, enhancing ROS production, and activating the NF-κB pathway, which contributes to metabolic disorders in PCOS patients (141). Additionally, HA promotes the secretion of and pro-inflammatory factors, subsequently suppressing folliculogenesis and ovulation, resulting in a cascade of events including pyroptotic death of ovarian GCs, follicular dysfunction, and ovarian interstitial cell fibrosis (15, 142). Increased endometrial cytokine synthesis and inflammation in PCOS patients induced by excess androgens through TLR4/IRF-7/NF-κB signaling contributes to inflammation in PCOS patients (143). Higher serum total testosterone and free testosterone index (FTI) were also found to be linked to an elevated risk of NAFLD in women with PCOS, independent of obesity and IR (106, 144, 145). HA induces ovarian inflammation in PCOS mice by activating the NLRP3 inflammasome, resulting in follicular dysfunction, ovarian fibrosis, and pyroptotic death (146). *In vitro* studies have indicated that DHEA directly inhibits the proliferation and promotes apoptosis of human ovarian granulosa tumor cell line (KGN) cells by down-regulating IFN-γ expression via the activation of the PI3K/AKT signaling pathway (147). DHEA administration directly activates the MNC cells and increases heightened sensitivity to glucose intake. In lean, healthy women, oral androgens increase mRNA expression of androgen receptors (AR) and stimulate TNF-α release from MNC in response to glucose-induced inflammation (148, 149).

#### 3.2.2 Progesterone

Progesterone, a critical anti-inflammatory steroid hormone, has recently been studied. Although the exact anti-inflammatory mechanism of progesterone remains unclear, current evidence suggests that its effects encompass both non-specific and specific immune regulation. The non-specific regulation is hypothesized to involve the inhibition of NF-κB activation, cyclooxygenase (COX) and prostaglandin synthesis. On the other hand, specific immune regulation is thought to include the modulation of T cell activation and cytokine production by immune cells (150). Notably, a study conducted on patients with COVID-19 demonstrated that progesterone exhibited therapeutic effects that are comparable to those of glucocorticoids in preventing severe illness and mortality associated with SARS-Cov2 infection (151–153). Furthermore, progesterone decreases the production of IL-1β, IL-6, TNF-α, and IL-12, as well as MCP-1/CCL2, suggesting it as a valuable adjunct to current SARS-CoV-2 treatment regimens (154). In PCOS patients, oligo/anovulation leads to reduced progesterone levels, which may result in inadequate inhibition of inflammation at the myometrium and maternal-fetal interface (155–158). This deficiency is associated with an increased risk of adverse pregnancy outcomes (159). Moreover, progesterone enhances the release of gonadotropin-releasing hormone (GnRH) and increases the sensitivity of the pituitary gland to GnRH, thereby triggering an LH surge. This results in the normalization of hyperandrogenemia and hyperinsulinemia levels, restore the physiological balance between androgens, estradiol, and progesterone within the menstrual cycle (160). Consequently, PCOS patients with progesterone deficiency may be at an increased risk of developing multi-system diseases owing to elevated systemic inflammation.

#### 3.2.3 Estrogen

Estrogen plays a dual role in regulating the immune system in women. During pregnancy, elevated estrogen levels inhibit the production of pro-inflammatory cytokines such as TNF-α, IL-1β, IL-6, MCP-1, iNOS, and MMPs. Concurrently, it reduces NK cell activity. Additionally, high concentrations of estradiol stimulate anti-inflammatory cytokines such as IL-4, IL-10, and TGF-β. This indicates that the sustained high levels of estrogen, in conjunction with progesterone, during gestation, play a synergistic role in suppressing the immune response and inflammation, which is crucial for the preservation of normal fetal development (161). In contrast, at the lower concentrations observed in non-pregnancy states, estrogen stimulates the production of inflammatory cytokines such as TNF-α, IFN-γ, and IL-1β, while also enhancing the activity of NK cells (161). Clinically, estrogen supplementation has shown anti-inflammatory and protective effects under certain conditions associated with chronic inflammation, such as osteoporosis, CVDs, and neurodegeneration during menopausal hormone replacement therapy (MHT) (162). Conversely, estrogen exerts pro-inflammatory effects in specific autoimmune diseases (AIDs), including rheumatoid arthritis (RA), systemic lupus erythematosus (SLE), highlighting estrogen as a risk factor for the increased incidence of various AIDs in women than in men (161, 163). PCOS patients may exhibit a lower average estrogen level owing to the absence of the fluctuations and peaks that typically occur during ovulation and the luteal phase in non-PCOS women with regular ovulation. Although estrogen levels can reach the pregnancy-like concentrations during ovulation in healthy individuals, the oligo/anovulation characteristic of PCOS leads to a deficiency in high estrogen levels, which may predispose individuals to excessive inflammation. Further investigations are necessary to clarify the potential role of estrogen in the regulation of chronic systemic inflammation associated with PCOS.

### 3.3 Intestinal microecological imbalance in PCOS

The gastrointestinal tract (GI) is inhabited by trillions of microorganisms, including bacteria, archaea, fungi, and viruses. Collectively known as the gut microbiome, these microorganisms interact with the external environment (such as nutrients), immune system of the human intestinal barrier, metabolic intermediates, and substances released from cells to establish the intestinal microecosystem (164). The gut microbiota, often referred to as the “second genome” in human beings, has significant clinical implications. Disturbances in the gut microbiota have been linked to various chronic health conditions such as metabolic syndrome, mental, and psychological diseases, and cancer (165–169). Gut microbiota disturbance is a major characteristic observed in PCOS patients and PCOS-like rodent models (170–172). Whole-genome shotgun sequencing demonstrated no significant difference in bacterial alpha diversity between PCOS patients and healthy controls; however, there was a significant increase in beta diversity in PCOS patients than that observed in healthy controls (173). DHEA-treated PCOS rats, exhibited a reduction in the relative abundances of Turicibacter, Anaerofustis and Clostridium sensustricto at the genus level. These findings underscore the potential role of gut microbiota in the pathogenesis and progression of PCOS.

**Figure 3:**
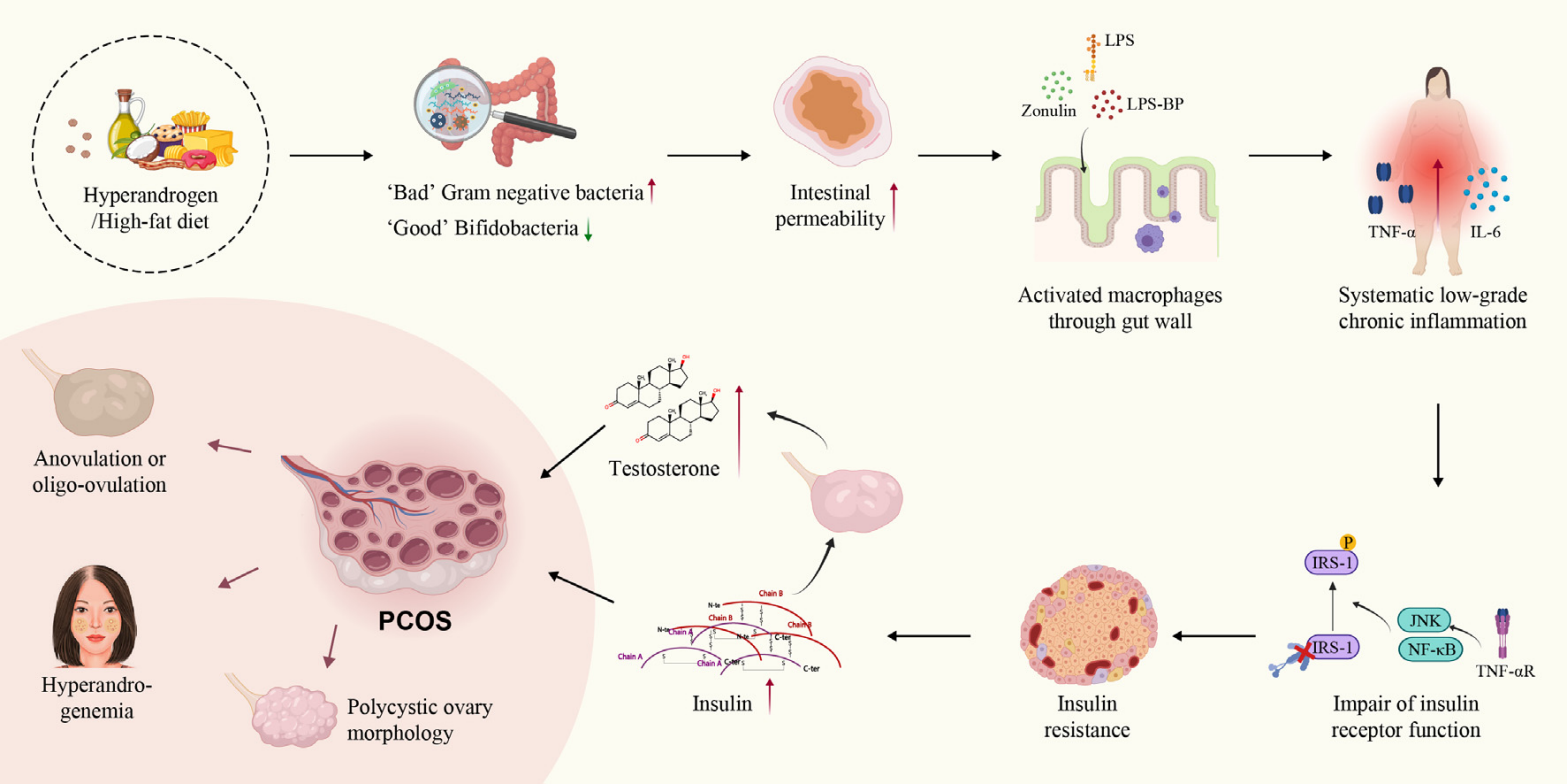
Theory of dysbiosis of gut microbiota in PCOS. Both hyperandrogenism and high-fat diet increase the proportion of harmful gut bacteria, leading to increased intestinal permeability and the release of LPS, LPS-BP, zonulin, and activated macrophages from the colonic lumen into the circulation. SLCI disrupts glucolipid metabolism and increases insulin and testosterone secretion. Finally, SLCI induced changes in ovarian morphology, and reproductive hormone imbalances in PCOS.

The interaction between the gut immune barrier and these microbes contributes to the GI tract becoming a potential source of chronic inflammation, which is closely associated with gut microecology (174, 175). In 2012, Tremellen et al. proposed the theory of Dysbiosis of Gut Microbiota (DOGMA) in the inflammatory pathogenesis of PCOS. They suggested that diet-induced imbalances between beneficial and harmful gut bacteria lead to increased intestinal permeability involving lipopolysaccharide (LPS), LPS-binding protein (LPS-BP), and zonulin entering systemic circulation, activating the TLR-4/NF-κB-mediated inflammatory response. This interference can affect insulin receptor function, causing IR, and potentially promoting testosterone synthesis in the ovaries, contributing to PCOS (176, 177). Studies have also consistently demonstrated that androgen exposure can cause intestinal dysbiosis, forming a vicious circle in PCOS patients (9, 178–180). Furthermore, the transplantation of androgen-induced gut microbiota into pseudo germ-free recipients disrupts glucolipid metabolism, ovarian morphology, and reproductive hormone imbalance (178) (**Figure 3**).

**Figure 4:**
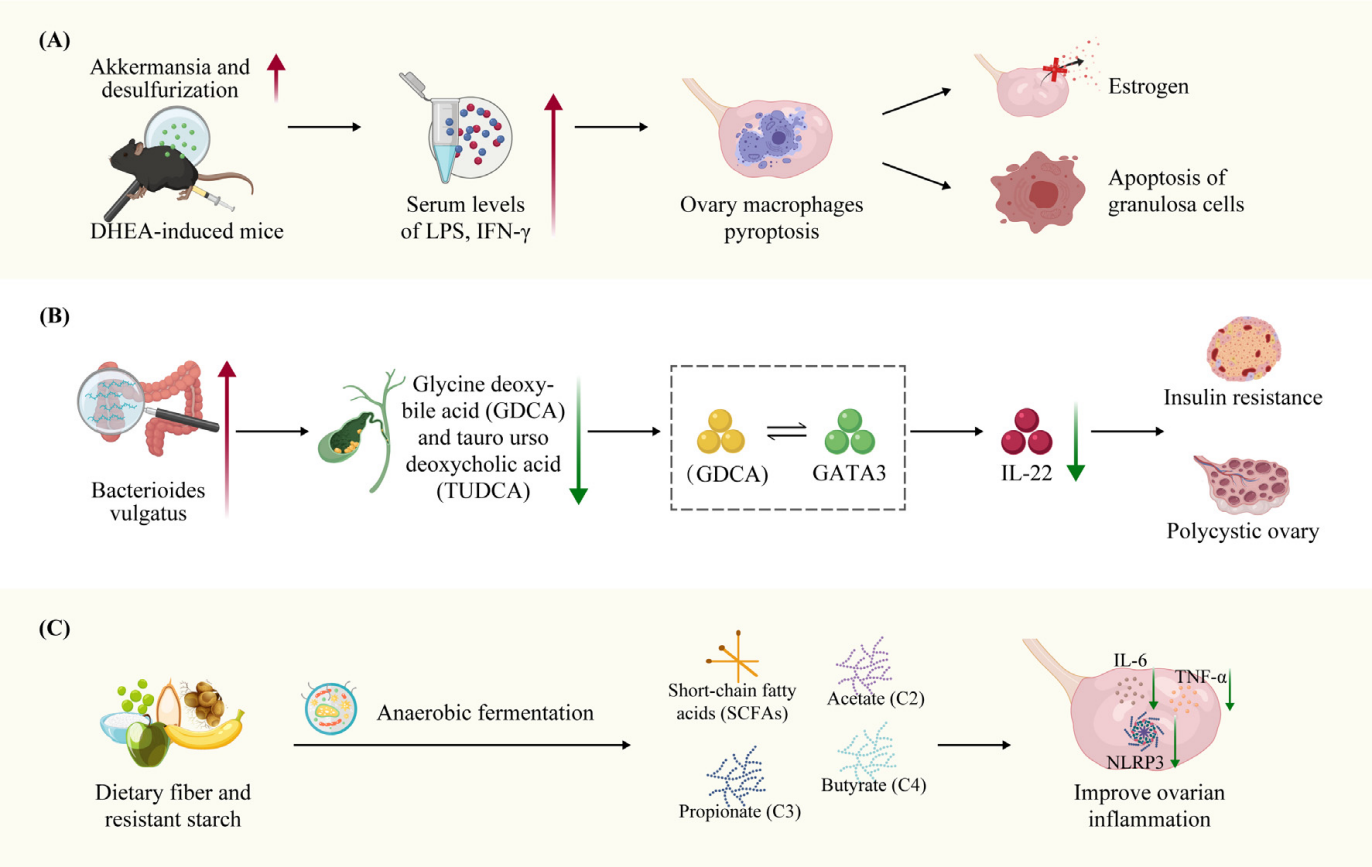
Dysbiosis of the gut microbiota and bile acid metabolites in PCOS ovarian function through inflammation. **(A)** Bacteroides vulgatus were significantly elevated levels in the intestinal microbiota of individuals with PCOS-like mice, accompanied by reduced concentrations of bile acid metabolites GDCA and TUDCA. GDCA stimulates IL-22 secretion via GATA-binding protein 3(GATA3). The Bacteroides vulgatus metabolite agmatine activates the farnesoid X receptor (FXR) pathway, leading to the inhibition of glucagon-like peptide-1 (GLP-1) secretion in intestinal epithelial L cells. This contributes to IR and ovarian dysfunction in PCOS-like mice. **(B)** Elevated abundance of Akkermansia and desulfurization gut bacteria, as well as increased serum levels of LPS and interferon IFN-γ, were observed in DHEA-induced PCOS-like mice. IFN-γ has the potential to induce pyroptosis in macrophages within the ovaries, ultimately resulting in abnormal ovarian function. **(C)** SCFAs have the potential to modulate ovarian inflammation by reducing pro-inflammatory IL-6, TNF-α and NLRP3 inflammasome secretion in the ovaries.

The impact of intestinal microbiome dysbiosis on the inflammatory processes that affect ovarian function in PCOS has been previously demonstrated. Huang et al. observed an increased abundance of Akkermansia and desulfurization bacteria in the intestines, as well as elevated serum levels of lipopolysaccharide (LPS) and interferon IFN-γ in DHEA-induced mice. Notably, IFN-γ can trigger pyroptosis in macrophage within the ovaries, which disrupts of estrogen production and promotes apoptosis of GCs, and ultimately leading to the abnormal ovarian function in PCOS mice. However, treatment with disulfiram and metformin increased the abundance of intestinal Akkermansia bacteria, decreased serum IFN-γ levels, and inhibited the pyroptosis in ovarian macrophages, thereby improving PCOS symptoms (39). Qiao et al. found that systemic inflammation in PCOS patients may be associated with an altered abundance of intestinal Bacteroides vulgatus and changes in metabolism of bile acids glycine deoxybile acid (GDCA) and tauroursodeoxycholic acid (TUDCA). The mechanism involves the interaction of the bile acid metabolite GDCA with the intestinal inherent (innate) group 3 lymphocytes GATA binding protein 3(GATA3), resulting in reduced secretion of the anti-inflammatory regulator IL-22.The therapeutic potential of IL-22 has been demonstrated and its administration shown to ameliorates IR, ovarian dysfunction, and infertility in PCOS (173). Furthermore, in DHEA-induced female mice exhibited upregulation of the metabolite agmatine from Bacteroides vulgatus was upregulated, which activates the farnesoid X receptor (FXR) pathway to inhibit glucagon-like peptide-1 (GLP-1) secretion in intestinal epithelial L cells. Furthermore, IL-8, IL-6, IL-1β and IL-18 were upregulated, contributing to ovarian inflammation. These findings suggested that the agmatine-FXR-GLP-1 signaling axis contributes to IR and ovarian dysfunction in PCOS-like mice (181). In addition to bile acid metabolites, short-chain fatty acids (SCFAs), primarily composed of acetate (C2), propionate (C3), and butyrate (C4), are beneficial metabolites derived from the fermentation of dietary fiber and resistant starch by the gut microbiota. Furthermore, SCFAs serve as potent anti-inflammatory modulators, capable of inducing Treg differentiation and interleukin secretion in peripheral tissues (182–184). Animal experiments have demonstrated that supplementation with SCFAs, such as butyric acid, can enhance ovarian function and reduce the levels of inflammatory factors within the ovaries. Butyric acid inhibits of m6A methyltransferase METTL3 expression in KGN cells, resulting in a decrease in FOSL2m6A methylation level and mRNA expression (185). Therefore, gut microbiota dysbiosis and the alterations in its metabolites contribute to the low-grade chronic inflammation present in the peripheral blood and ovaries of PCOS patients (**Figure 4**).

### 3.4 Vitamin D and selenium deficiency in PCOS

Vitamin D and selenium are involved in the regulation of immunity and inflammation, and deficiencies in these essential substances are associated with an increased risk of PCOS (186). Multiple case-control studies have reported lower concentrations of 1,25-dihydroxyvitamin D, the active form of vitamin D, in women with PCOS patients (187–191). Approximately 67%-85% of PCOS patients either exhibit vitamin D deficiency or insufficiency (192), which is more prevalent in PCOS patients with HA (193). In addition to its role in regulating calcium and phosphate metabolism and maintaining skeletal structure, vitamin D also exhibits anti-inflammatory effects. Vitamin D receptors and the enzymes responsible for activating vitamin D are expressed in both innate and adaptive immune cells, including monocytes, macrophages, dendritic cells, and lymphocytes. These immune cells not only facilitate the secretion of vitamin D but also respond to activated vitamin D through the autocrine pathways (194, 195). Vitamin D deficiency has been implicated in systemic inflammation and the pathogenesis of PCOS. A meta-analysis has shown the administration of vitamin D in PCOS patients can reduce the levels of inflammatory mediators, such as serum hs-CRP, serum total testosterone (TT), and oxidative stress indices, while improving the overall antioxidant capacity. Furthermore, vitamin D replacement therapy may have a beneficial effects on IR, hormone regulation, menstruation, and ovulation disorders in PCOS patients (196–199).

A systematic review reported that plasma selenium levels were lower in PCOS patients than in healthy controls in two out of three case-control studies, with serum selenium levels were negatively correlated with androgen levels in PCOS patients. Selenium has shown therapeutic benefits owing to the its immunomodulatiory properties (200–202). Selenium, particularly the amino acid selenocysteine, exerts its biological effects primarily in the form of selenoproteins. At least 25 selenoproteins have been identified in human beings, with glutathione peroxidase (GPX) being the primary selenoprotein responsible for regulating excessive free radical production at the sites of inflammation. Apart from GPX, other selenoproteins have been recognized, including selenin-S, which modulates inflammatory cytokines, and selenin-P, which acts as an inducer of homeostasis (203–205). Selenoproteins play a crucial role in regulating inflammation and modulating clinical outcomes in various diseases including cancer, diabetes, Alzheimer’s disease, mental disorders, CVDs, fertility disorders, inflammation, and infections, including SARS-CoV-2 (206). Studies conducted on PCOS-like rat models have revealed that treatment with selenium nanoparticles, either alone or in combination with metformin, can restore the estrus cycle, reduce blood glucose, and insulin levels, improve hyperlipidemia, reduce serum testosterone levels and enhanced ovarian histopathology, accompanied by decreased levels of serum pro-inflammatory factors TNF-α and IL-6 as well as oxidative stress biomarkers MDA and GPX in the ovarian tissues (207–209). Although these animal studies have demonstrated the effectiveness of selenium in PCOS treatment. A recent systematic review concluded that selenium supplementation only positively affected total antioxidant capacity (TAC) in PCOS patients without significantly improving BMI, body weight, LDL, HDL, triglyceride, total testosterone, HOMA-IR, NO, glutathione(GSH), MDA, and FPG levels (210). Further clinical randomized controlled clinical trials are necessary to confirm the efficacy and safety of selenium supplementation or selenium-based drugs for treating of PCOS.

### 3.5 HHcy in PCOS

Preliminary investigations in observational and randomized controlled trials have indicated that women with PCOS exhibit higher serum homocysteine (Hcy) levels, along with higher levels of markers of CVDs, such as hs-CRP, soluble CD40 ligand (sCD40L), and asymmetric dimethylarginine (ADMA) than did healthy controls (211, 212). In atherosclerosis, Hcy acts as a vascular pro-inflammatory cytokine capable of activating the monocyte-macrophage system (213). Hcy stimulates the release of MCP-1 and IL-8 from peripheral blood monocytes, which are two major chemokines involved in leukocyte trafficking (214). Additionally, Hcy induces the expression and secretion of IL-8 by aortic endothelial cells and stimulates MCP-1 production by vascular smooth muscle cells (215, 216). Accumulation of MCP-1 and IL-8 has also been observed in macrophages from human atherosclerotic plaques. Furthermore, even slightly elevated levels of Hcy (10 μmol/L) can effectively stimulate the accumulation of MCP-1 and IL-8 in the injured artery wall, thereby promoting macrophage-mediated inflammation and atherosclerosis (217, 218). These studies suggested that Hcy primarily targets the monocyte-macrophage interaction.

A cross-sectional study revealed a positive correlation between HHcy and IR in PCOS patients. Compared with individuals with normal Hcy levels, individuals within the HHcy group exhibited increased numbers of CD14^++^CD16^+^ monocytes and higher levels of IL-1β, IL-6 and IL-2 in the peripheral blood in PCOS patients. These results suggested that the activation of inflammatory monocytes may be related to the Hcy-induced PCOS-IR phenotype (219). Moreover, Mondal et al. reported that female rats exposed to Hcy developed a PCOS-like reproductive-endocrinal phenotype associated with lipid metabolism disorders without involvement of other modalities used for PCOS modeling. Furthermore, this study demonstrated that HHcy and HA share a common mechanism involving the disruption of PCSK9-LDLR pathway leading to lipid homeostasis disturbances in PCOS (220). Another study showed that HHcy promotes IR and adipose tissue inflammation in PCOS mice by reducing estrogen production while inhibiting the polarization of M2 anti-inflammatory macrophages (221). This suggests that HHcy may also contribute to chronic inflammation-related metabolic disorders, potentially through its influence on monocyte-macrophage activity.

## 4 Intervention

### 4.1 Non-Pharmacological Management of PCOS

International evidence-based guidelines recommend non-pharmacological lifestyle management, including dietary modifications and regular physical activity, as the first-line treatment for infertility associated with PCOS to optimize health generally and improve fertility outcomes (222).

#### 4.1.1 Diet

A healthy diet is the cornerstone of a healthy lifestyle and can significantly ameliorate the symptoms of intestinal dysbiosis, inflammatory status, and reproductive and metabolic abnormalities in PCOS. A clinical study reported that the very low-calorie ketogenic diet (VLCKD) improved ovarian function in obese women with PCOS. In this study, 25 obese patients with PCOS were enrolled to receive a VLCKD intervention for 12 weeks. Significant reductions in BMI, waist circumference (WC), and HOMA were observed. These results suggest that VLCKD may be an effective strategy for ameliorating metabolic and ovulation dysfunction in women with PCOS (223).

The Mediterranean diet (MD), known for its anti-inflammatory properties, has shown promise in managing PCOS. Barrea et al. conducted a case-controlled cross-sectional study that revealed that high adherence to the MD diet resulted in lower CRP levels, HoMA-IR, testosterone levels, and Ferriman-Gallwey scores. This preliminary evidence suggests that the MD diet reduces disease severity, IR, and hyperandrogenemia in PCOS (224).

Intermittent fasting has emerged as a viable approach for reducing weight and energy intake. The forms of intermittent fasting include three diets: alternate-day fasting (ADF), 5:2 diet, and time-restricted eating (TRE). A clinical study reported 15 anovulatory PCOS patients aged 18–31 years who completed 8-h time-restricted feeding for 6-weeks experienced substantial improvements. After TRE, body weight, BMI, HOMA-IR, and hs-CRP decreased, whereas SHBG and insulin-like growth factor 1(IGF-1) levels increased, and irregular menstrual cycles improved in 73.3% (11/15) of patients (225).

These results underscore the potential of a healthy diet in reducing weight and body fat and improving menstruation, hyperandrogenemia, IR, and chronic inflammation in PCOS patients.

#### 4.1.2 Nutrient Supplementation

Nutrients, mainly including vitamins (such as vitamin D), vitamin-like nutrients (such as α-lipoic acid), and minerals (such as magnesium), are essential components of a healthy diet. Nutrient supplementation can have a positive impact on PCOS patients (226). A randomized double-blind placebo-controlled clinical trial conducted by Bahmani et al. demonstrated that folic acid supplementation significantly reduced the plasma levels of Hcy, HOMA-B, hs-CRP, and MDA and significantly increased the plasma levels of TAC and GSH. These findings suggest that folic acid supplementation has a potential clinical role in improving metabolic conditions and reducing inflammation and oxidative stress in PCOS patients (227). In a randomized, double-blind, placebo-controlled trial involving patients with PCOS, 60 participants were randomly assigned to two groups (n = 30 in each group), one receiving a supplement of 1000 mg omega-3 and 400 IU of vitamin E, and the other receiving a placebo for a duration of 12 weeks. The study results indicated that the combined supplementation of omega-3 and vitamin E significantly reduced CIMT and serum hs-CRP levels (228). In a clinical study conducted by Stracquadanio et al., continuous administration of myo-inositol, gymnemic acid, and L-methylfolate for 6 months demonstrated substantial beneficial effects in PCOS patients, including improvement in menstrual cycle regularity and metabolic parameters, reduction in BMI and total testosterone, and increased insulin sensitivity (229). Hager et al. conducted a randomized controlled trial investigating the effects of a standardized micronutrient supplementation in PCOS patients. The supplement included omega-3 fatty acids, folic acid, selenium, vitamin E, catechin, glycyrrhizin, and coenzyme Q10, which was administered for 3 months. The study demonstrated that compared with the control group, the group receiving the micronutrient supplement experienced a significant decrease in the LH/FSH ratio, testosterone, and AMH levels (230). These findings indicated that nutrient supplementation may be beneficial for ameliorating some of the adverse health outcomes associated with PCOS. Specifically, nutritional supplements can potentially improve menstrual cycle regularity, IR, inflammation, and oxidative stress in PCOS patients.

#### 4.1.3 Physical Activity

Physical activity is recommended as a first-line approach for managing PCOS, particularly for overweight or obese women. The guidelines recommend a minimum of 150 min/week of moderate-intensity exercise, 75 min/week of vigorous-intensity exercise, or a combination of both (231). A meta-analysis by Moori et al. demonstrated that exercise training effectively lowered inflammatory markers, such as serum CRP levels (232). In a randomized clinical trial, high-intensity interval training (HIIT) elicited greater improvements in cardiometabolic and reproductive outcomes than moderate-intensity interventions in overweight women with PCOS. Patten et al. further found that HIIT considerably improved the oxygen-carrying capacity of blood than did moderate-intensity exercises. HIIT substantially increased SHBG levels and regularized menstrual cycles in PCOS patients (233). In summary, engaging in regular physical activity, particularly HIIT, is beneficial for women with PCOS, as it not only helps in managing weight but also has positive effects on inflammation, cardiometabolic health, and reproductive function. These findings emphasize the importance of incorporating exercise into the treatment plan for PCOS, highlighting its potential to improve the overall health and quality of life of affected individuals.

### 4.2 Pharmacological Interventions

#### 4.2.1 Metformin

Metformin, a biguanide, improves insulin sensitivity, reduces androgen levels, and enhances oligo-amenorrhoea and subfertility in women with PCOS (234). Metformin increases gut Akkermansia abundance, reduces serum IFN-γ level released from T cells, and inhibits macrophage pyroptosis in ovaries in PCOS mice (234). Xue et al. revealed metformin alleviated PCOS by modulating gut microbiota, reducing plasma LPS levels, and decreasing the plasma and ovarian levels of inflammatory cytokines, including TNF-α, IL-6, and IL-17A, in PCOS patients (235).

#### 4.2.2 Traditional Chinese Medicine

Traditional Chinese Medicine (TCM) formulations and their active ingredients have been widely used to treat various gynecological diseases, including PCOS. Herbal medicines and active ingredients regulate gut microbiota composition and reduce systemic and ovarian inflammation in PCOS-like models (236–238). Wang et al. reported the TCM decoction Bu Shen Hua Zhuo formula (BSHZF) administration improved gut microbiota function in rats with letrozole-induced PCOS and inhibited the activation of the TLR4/NF-kB signaling pathway in PCOS-related ovarian tissue, decreasing the pro-inflammatory cytokines TNF-a, IL-6, and IL-8 (236). Zhu et al. reported Gui-zhi-Fu-ling Wan treatment reduced inflammatory markers such as hs-CRP, IL-6, and TNF-α and improved PCOS-IR by remodeling the relative abundance of multiple intestinal flora (237). Chang et al. reported that Shaoyao-Gancao Decoction (SGD), commonly used to treat multiple gynecological disorders such as dysmenorrhea, adenomyosis, and PCOS, modulated gut microbiota composition, and alleviated chronic low-grade inflammation by downregulating cytokines including IL-18, IL-1β, IL-6, and TNF-α in both serum and ovarian mRNA expression in PCOS rats (238). In summary, these studies underscore the therapeutic potential of TCM for treating PCOS by targeting gut microbiota and inflammatory pathways, which may offer a complementary approach to conventional treatments. Modulation of the gut microbiota using TCM formulations holds promise as a therapeutic strategy for PCOS.

#### 4.2.3 Other Pharmacological Interventions

In clinical practice, other pharmacological interventions for PCOS include combined oral contraceptives (COCs) and antiandrogens. COCs are commonly prescribed to PCOS patients with menstrual irregularities and clinical hyperandrogenism. COCs reduce free testosterone levels by increasing SHBG production in the liver, thereby alleviating hyperandrogenism (239). Anti-androgens such as finasteride, flutamide, spironolactone, or bicalutamide, along with lifestyle modifications, are more effective in improving hirsutism, SHBG, fasting insulin, and the fasting insulin:glucose ratio. Current evidence does not support the preferential use of anti-androgens over COCs for the treatment of hyperandrogenism in PCOS. However, antiandrogens are not preferred over COCs for hyperandrogenism unless COCs are contraindicated or ineffective (240). Moreover, low-dose spironolactone in PCOS rats reduces the oxidative stress markers (MDA) and inflammatory biomarkers such as NF-kB, TNF-α, and IL-6 (241).

## 5 Conclusion and perspectives

In conclusion, SLCI plays a critical role in the pathogenesis and progression of PCOS, contributing to the manifestation of multiple symptoms and an increased risks of various long-term complications associated with PCOS. Chronic systemic inflammation observed in PCOS patients is linked to an imbalance between pro-inflammatory and anti-inflammatory intrinsic mechanisms. Targeted inflammatory regulation therapy may be an effective approach for alleviating PCOS phenotypes of PCOS and improving patient outcomes.

As for a test panel that should become standard in diagnosing PCOS patients at SLCI state, while there is no consensus on a specific “standard test panel” for diagnosing SLCI in PCOS patients, the mentioned markers could be part of a comprehensive diagnostic approach. It is important for healthcare providers to consider the individual patient’s symptoms and risk factors when determining the appropriate tests to order. Therefore, a potential test panel could include hs-CRP,IL-1 Rα,IL-6,IL-17 E/IL-25,IL-17A,IL-18,TNF-α,MIP-1α,and other markers that reflect the inflammatory state and metabolic health of the PCOS patients (242, 243).

International evidence-based guidelines recommend non-pharmacological lifestyle management, such as the ketogenic diet, the Mediterranean diet, intermittent fasting, and regular physical activity, as the first-line treatment for infertility in PCOS. These pharmacological approaches aim to reduce inflammation and optimize overall health to improve fertility (231, 244). Regarding pharmacological management of PCOS, periodic use of progesterone, including the use of COCs, can effectively regulate menstruation in PCOS patients, lower androgen levels, and protect the endometrium. Additionally, progesterone has potential to control chronic inflammation (150, 245). Metformin is an excellent regulator of inflammation and plays an important role in the treatment of PCOS by improving the metabolic disorders, reducing androgen levels and promoting follicular development and ovulation (246–248). Xue et al. found that metformin alleviates PCOS by modulating gut microbiota, resulting in reducing plasma LPS levels along with decreased plasma and ovarian inflammatory cytokines TNF-α, IL-6, and IL-17A levels in PCOS patients (235). Furthermore, statins, as antihyperlipidemic drugs, can reduce HA, improve lipid profiles, and reduce systemic inflammation in women with PCOS (249). Additionally, curcumin, inositol, CoQ10, and microelement selenium, and vitamin D is widely recommended as a fundamental intervention for PCOS owing to their ability to reduce IR and inflammation, enhance ovarian function restoration, restore hormonal balance, and regulate the menstrual cycle in PCOS. Other anti-inflammatory and antioxidant dietary supplements, such as folic acid, inositol, vitamin E, omega-3 fatty acids, alpha lipoic acid, N-acetylcysteine, have shown potential adjuvant therapeutic effects by ameliorating IR, lipid profile, reducing inflammation and oxidative stress markers of PCOS (227–229, 250–255).

Treatment options to relieve gut dysbiosis in PCOS patients include innovative approaches like fecal bacteria transplantation and “prebiotics,” which aim to improve intestinal microecology (256). Additionally, drugs targeting inflammatory cytokines have been found to ameliorates PCOS-related phenotypes. For example, Lang et al. conducted a study on the TNF-α inhibitor etanercept (ETA), which inhibited serum testosterone levels, TNF-α and MCP-1 levels, decreased excessive recruitment of lipid droplets, altered levels of pre-adipose differentiation markers, and abnormal development of follicles in letrozole-induced PCOS rat models. This suggests that anti-TNF-α therapy with ETA may have a potential ameliorative effect associated with its ability to reduce excessive androgen levels on PCOS (257). Moreover, some TCM or ingredients of Chinese herbal medicines, such as the BSHZF, Guizhi Fuling Wan, and SGD improved IR and ameliorated sex hormone disturbances in PCOS through anti-inflammatory effects activation of the PI3K/AKT pathway, and modulation of gut microbiota (236–238). In conclusion, although the etiology of PCOS remains unclear, given the important role of SLCI in PCOS, comprehensive treatment strategies involving long-term management, including non-pharmacological lifestyle interventions combined with pharmacological approaches aimed at ameliorating inflammation, should be adopted to ultimately improve the clinical phenotype of PCOS, reduce the incidence of long-term complications, and enhance overall health among individuals with PCOS.
